# The Role of Neutrophil Myeloperoxidase in Models of Lung Tumor Development

**DOI:** 10.3390/cancers6021111

**Published:** 2014-05-09

**Authors:** Amy L. Rymaszewski, Everett Tate, Joannes P. Yimbesalu, Andrew E. Gelman, Jason A. Jarzembowski, Hao Zhang, Kirkwood A. Pritchard, Haris G. Vikis

**Affiliations:** 1 Department of Pharmacology and Toxicology and MCW Cancer Center, Medical College of Wisconsin, Milwaukee, WI 53226, USA; E-Mails: arymaszewski@mcw.edu (A.L.R.); etate@uwm.edu (E.T.); jyimbesalu@mcw.edu (J.P.Y.); 2 Department of Surgery, Washington University in St. Louis, St. Louis, MO 63130, USA; E-Mail: gelmana@wudosis.wustl.edu; 3 Department of Pathology, Medical College of Wisconsin, Milwaukee, WI 53226, USA; E-Mail: jjarzemb@mcw.edu; 4 Department of Surgery and MCW Cancer Center, Medical College of Wisconsin, Milwaukee, WI 53226, USA; E-Mails: hzhang@mcw.edu (H.Z.); kpritch@mcw.edu (K.A.P.)

**Keywords:** neutrophils, myeloperoxidase, KYC, lung, tumor

## Abstract

Chronic inflammation plays a key tumor-promoting role in lung cancer. Our previous studies in mice demonstrated that neutrophils are critical mediators of tumor promotion in methylcholanthrene (MCA)-initiated, butylated hydroxytoluene (BHT)-promoted lung carcinogenesis. In the present study we investigated the role of neutrophil myeloperoxidase (MPO) activity in this inflammation promoted model. Increased levels of MPO protein and activity were present in the lungs of mice administered BHT. Treatment of mice with *N*-acetyl lysyltyrosylcysteine amide (KYC), a novel tripeptide inhibitor of MPO, during the inflammatory stage reduced tumor burden. In a separate tumor model, KYC treatment of a Lewis Lung Carcinoma (LLC) tumor graft in mice had no effect on tumor growth, however, mice genetically deficient in MPO had significantly reduced LLC tumor growth. Our observations suggest that MPO catalytic activity is critical during the early stages of tumor development. However, during the later stages of tumor progression, MPO expression independent of catalytic activity appears to be required. Our studies advocate for the use of MPO inhibitors in a lung cancer prevention setting.

## 1. Introduction

Lung cancer remains the major cause of cancer-related death in the Western World [1,2,3,4,5]. While surgical resection is effective for those with early stage disease, the majority of patients present with advanced malignancies requiring systemic and cytotoxic treatments such as chemotherapy, which have limited efficacy. Consequently, chemoprevention by medicines tailored to individuals at high-risk for lung cancer (e.g., underlying inflammatory disease, heavy smokers, familial history) remains a promising disease control alternative. Identification of molecular targets of prevention and evaluation of non-toxic agents in pre-clinical animal models of lung cancer is a necessary prerequisite to chemoprevention in humans.

As early as the 19th century it was observed that chronic inflammation in certain tissues predisposes one to cancer [6,7,8,9]. Prolonged inflammation can induce DNA damage due to increased exposure to oxidative stress [10]. To model the role of lung inflammation in tumorigenesis, a 2-stage approach is often used. This involves single administration of the tobacco-related carcinogen 3-methylcholanthrene (MCA) to initiate tumorigenesis, followed by multiple administrations of the lung injury agent butylated hydroxytoluene (BHT) to promote tumorigenesis. BHT is non-carcinogenic and causes epithelial necrosis, resulting in compensatory proliferation of airway epithelial cells and significant leukocyte infiltration into the lung [11,12,13,14,15,16]. The MCA/BHT model is a widely accepted tumor promoter and model of lung injury [11,15,17,18]. Using cell depletion studies, we previously reported that neutrophils (and not T lymphocytes) are critical mediators of lung tumor promotion by BHT [19]. Others have demonstrated that inhibition of IL8 receptor-dependent neutrophil recruitment impedes tumor formation in inflammation-driven cancer models of the colon and skin [20]. These observations mirror the association of lung neutrophilia with increased risk of lung cancer as seen in chronic obstructive pulmonary disorder (COPD) [21,22,23,24,25]. In humans, elevated numbers of neutrophils in bronchoalveolar lavage fluid (BALF), blood, or tumor tissue from lung cancer patients is prognostic for poor survival [6,26,27,28,29,30,31,32,33]. A major gap in our understanding remains the identification of the neutrophil specific activities that mediate tumor formation and growth. 

Neutrophils contain numerous substances used to defend against pathogens and to mediate wound healing in tissue [34,35]. Some of the neutrophil cytoplasmic granule components have been proposed to contribute to tumor proliferation, angiogenesis and metastasis [28,29]. One of these is myeloperoxidase (MPO), a heme peroxidase enzyme that neutrophils express and secrete, that generates reactive oxygen/nitrogen species (ROS/RNS) as a mechanism for pathogen removal. Upon neutrophil activation, primary granules containing MPO can fuse with the plasma membrane to secrete contents into the extracellular milieu. During the respiratory burst MPO utilizes H_2_O_2_ to produce hypochlorous acid (HClO) that reacts with proteins, unsaturated fatty acids and any oxidizable group, to induce protein adducts and genetic mutations, and influence signaling pathways [36,37,38,39,40,41,42,43,44]. It has been suggested that extended exposure to ROS due to chronic inflammation results in DNA damage and genomic instability leading to malignant cell transformation [45,46]. A study in lung epithelial cells demonstrated that MPO can be internalized by the cells resulting in increased DNA damage [47]. Interestingly, prior studies have indicated that a single nucleotide polymorphism in the human MPO gene promoter region is associated with reduced MPO levels and lower risk for developing lung cancer [48]. Furthermore, patients suffering from COPD and lung cancer exhibit increased levels of MPO in serum and BALF [49,50]. Therefore, we hypothesized that MPO is a target for prevention of lung cancer and investigated its role in animal models of lung tumor development.

In our study, we observed increased levels of MPO in the lungs of BHT-treated mice, consistent with that observed in COPD and lung cancer patients [50]. We demonstrate that treatment with KYC, a novel tripeptide MPO inhibitor, reduced BHT-promotion of MCA-induced lung carcinogenesis by 50%. To our knowledge this study is the first demonstration for use of an MPO inhibitor in a mouse model of lung cancer. Interestingly, this MPO inhibitor had no effect in a tumor graft model in mouse, however the growth of the tumor graft was slowed in an MPO-knockout mouse, suggesting possible activity-independent effects of MPO on established tumor growth. That said, our studies suggest that use of MPO inhibitors in early in tumor development is a potential new strategy for lung cancer prevention.

## 2. Results and Discussion

### 2.1. BHT Administration Enhanced Neutrophil and Myeloperoxidase Levels in the Lung

**Figure 1:**
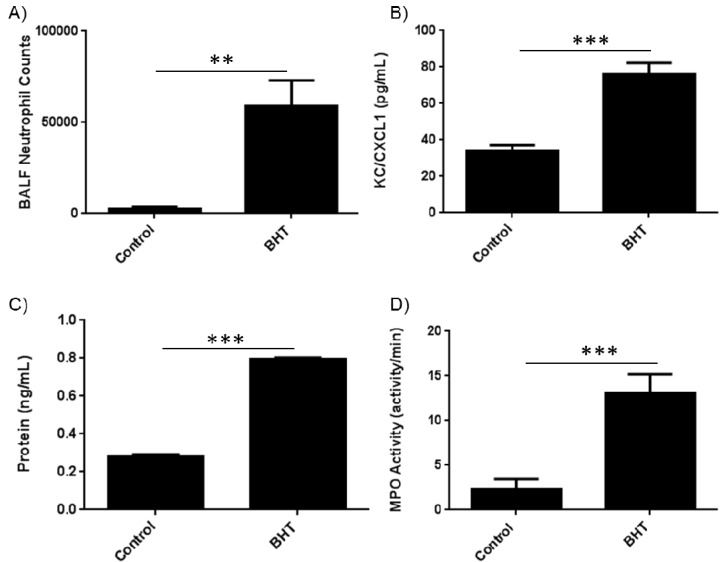
BHT-induced lung inflammation increases lung levels of neutrophils and peroxidase activity. Balb/cByJ mice were treated with 300 mg/kg BHT i.p. and euthanized three days post injection. (**A**) BALF was collected, stained for neutrophils (Ly6G^+^ Gr1^+^) and analyzed by flow cytometry. BHT caused an increase in the number of neutrophils collected in the BALF; (**B**) Neutrophil chemokine CXCL1/KC levels increased in mice treated with BHT; (**C**) BALF supernatant demonstrated increased protein levels in mice treated with BHT; (**D**) BHT enhanced BALF peroxidase activity in mice treated with BHT. NS, not significant; *, *p* < 0.05; **, *p* < 0.01; ***, *p* < 0.001; ****, *p* < 0.0001.

Our previous studies demonstrated a key role for neutrophils in inflammation-promoted lung tumorigenesis [19]. We therefore wanted to determine the neutrophil-related activities that mediate this effect. Consistent with previous studies we observed an increase in neutrophil numbers and the neutrophil chemoattractant, keratinocyte-derived cytokine (KC)/chemokine (C-X-C motif) ligand 1 (Cxcl1), in bronchoalveolar lavage fluid (BALF) from female BALB/cByJ (BALB) mice three days after treatment with BHT (Figure 1A,B). Protein exudates were similarly increased as measured in the BALF with BHT treatment, which is a hallmark of the inflammatory response (Figure 1C). Having demonstrated an increase in neutrophils in the BALF after BHT administration, we wanted to determine whether myeloperoxidase, a previously reported and highly expressed protumorigenic primary granule protein, was elevated in BALF post-BHT. We determined that there was a significant 6-fold increase in MPO activity after BHT administration (Figure 1D). This observation suggests that increased MPO activity is associated with lung inflammation in our tumor model, and justifies investigation of the use of MPO inhibitors in preventing tumor promotion. 

### 2.2. KYC Inhibited BHT-Induced MPO Activity, but not Infiltration of Neutrophils and Levels of MPO Enzyme in the Lung

KYC is a unique tripeptide inhibitor of myeloperoxidase and a potential *in vivo* preventive agent [51,52]. We subsequently tested whether KYC inhibited BHT-induced peroxidase activity in BALF. BALB mice were administered KYC (0.3mg/kg/d, subcutaneous) prior to administration of BHT. BALF was collected, and peroxidase activity was determined in the cell-free fraction. 

**Figure 2:**
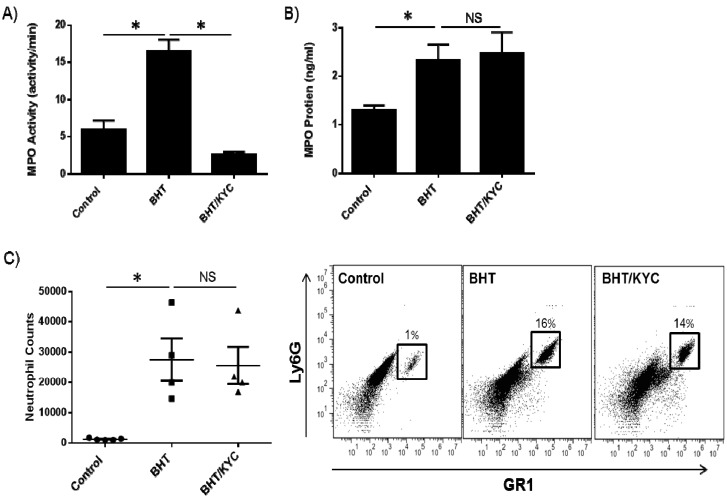
The MPO inhibitor *N*-acetyl lysyltyrosylcysteine amide (KYC) inhibits lung peroxidase activity and not neutrophil lung infiltration. (**A**) Mice were treated with 0.3 mg/kg of KYC via subcutaneous injection for one week prior to BHT administration (300 mg/kg) and treatment continued for 3 days until mice were euthanized. BALF was collected and BHT-stimulated peroxidase activity was reduced by KYC; (**B**) KYC did not affect BHT-induced levels of MPO protein in the BALF; (**C**) KYC treatment similarly did not reduce BHT-stimulated neutrophil levels (left panel). Representative flow cytometry plots are shown (right panel).

Peroxidase activity in BALF was significantly inhibited in the KYC treatment group (Figure 2A). KYC treatment did not affect BHT-induced MPO protein levels in BALF as determined by ELISA (Figure 2B). KYC is a reversible inhibitor of MPO and does not irreversibly damage the enzyme as compared to other suicide substrate inhibitors [51,52]. Whole BALF was stained for neutrophils using specific markers of Ly6G and Gr1. Correspondingly, BHT-induced stimulation of neutrophil numbers in the BALF was unaffected by KYC treatment (Figure 2C). 

### 2.3. KYC Inhibited BHT-Promoted Lung Carcinogenesis

We next tested the ability of KYC to block inflammation-promoted lung tumorigenesis in the MCA/BHT mouse model. Previous studies from our lab demonstrated that antibody-mediated depletion of neutrophils reduced lung tumor burden in the MCA/BHT model by 70% [19]. We therefore administered KYC (0.3 mg/kg/d, subcutaneous) to BALB strain mice beginning 4 days post-MCA treatment, and ending 1 week after the final BHT injection. 

**Figure 3:**
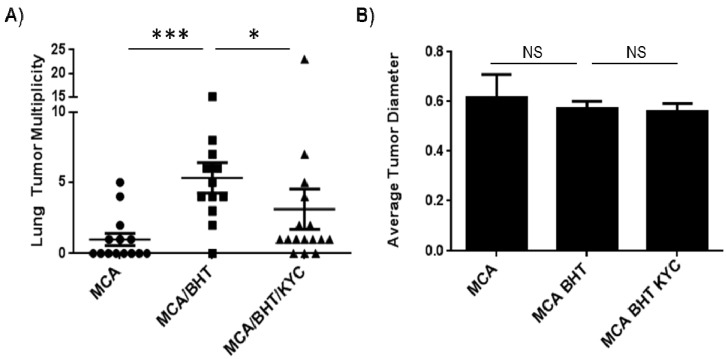
KYC reduces tumor multiplicity in an inflammation-promoted model of lung carcinogenesis. (**A**) BHT induced a 5-fold increase in lung tumor multiplicity (MCA: 1.0 ± 0.4; MCA/BHT: 5.3 ± 1.0). Lung tumor multiplicity was reduced by 50% in mice treated with KYC (3.1 ± 1.4); (**B**) Tumor size (*i.e.*, diameter) was unchanged between treatment groups.

The average number of surface lung tumors was promoted from 1.0 ± 0.4 to 5.3 ± 1.0 tumors/mouse by BHT (*p* < 0.001), which is consistent with our prior studies [19]. Administration of KYC reduced surface lung tumor multiplicity by 50% in BHT treated mice from 5.3 ± 1.0 to 3.1 ± 1.4 tumors/mouse (*p* < 0.05) (Figure 3A). Average tumor diameter was not significantly reduced by KYC treatment (Figure 3B), which is consistent with our prior neutrophil-depletion studies [19]. This demonstrates that KYC treatment shortly after tumor initiation and throughout inflammation-mediated tumor promotion is sufficient to block tumor multiplicity at 20 weeks.

### 2.4. KYC Did Not Inhibit Tumor Growth in a Heterotopic Syngeneic Graft Model of Lung Cancer

We next sought to understand the role of MPO in the *in vivo* growth of a heterotopic xenografted lung cancer cell line in mouse. Wild type female C57BL/6J (B6) or homozygous MPO knockout mice (MPO^–/–^) were given a subcutaneous injection of one million Lewis Lung Carcinoma (LLC) cells in their right flank.

**Figure 4:**
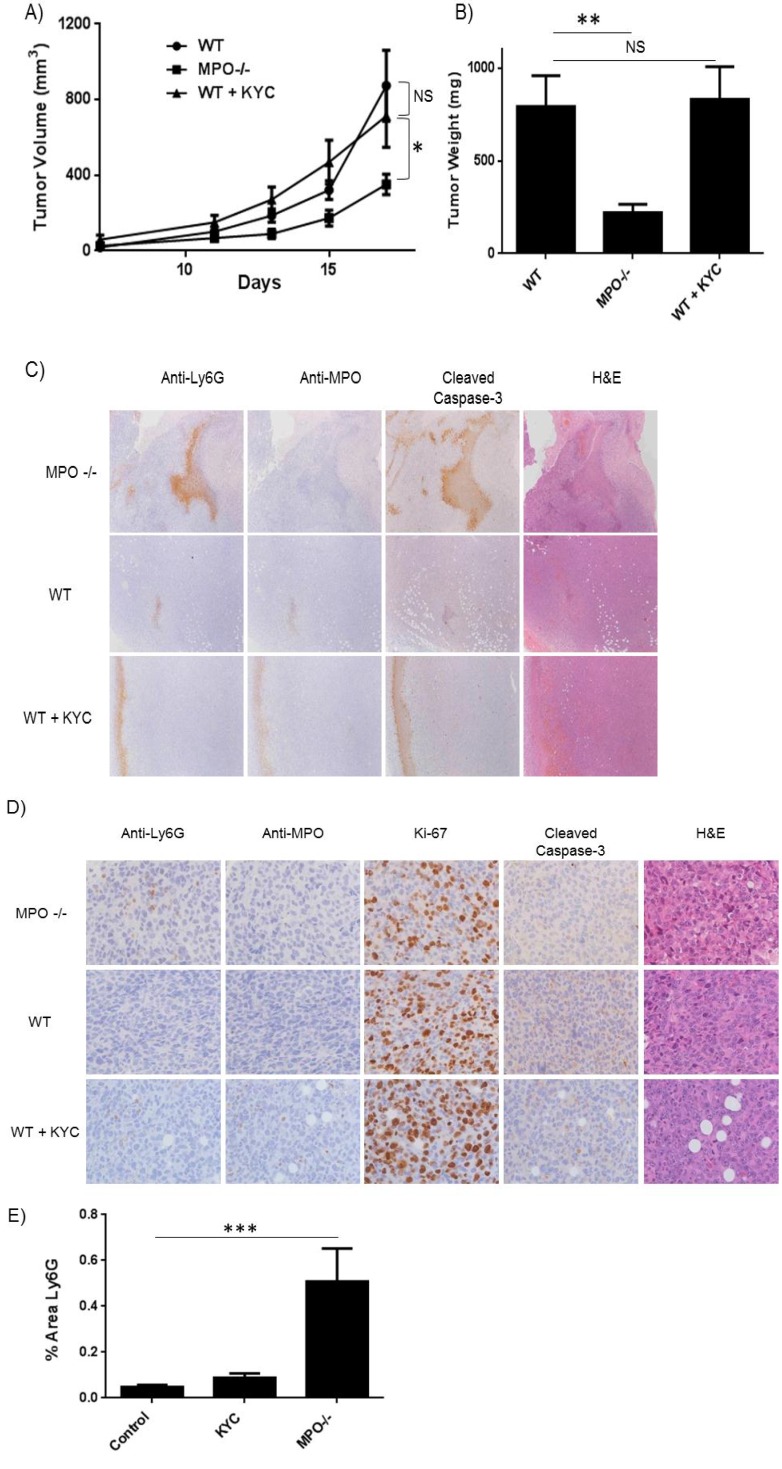
Genetic deletion of MPO slows tumor growth in a heterotopic syngeneic tumor model. WT and MPO^–/–^ C57BL/6J mice were injected in the right flank with Lewis Lung Carcinoma cells. (**A**) Tumor volume was monitored over the course of 17 days. MPO^–/–^ mice exhibited smaller tumors compared to WT. Wild type mice treated with KYC were no different than PBS treatment; (**B**) Tumors were surgically resected and weighed on day 17. The weight of tumors from MPO^–/–^ mice was significantly smaller compared to WT mice; (**C**) Representative images of tumors stained for Ly6G, MPO and apoptosis marker cleaved caspase-3 (4×); (**D**) Representative images of non-necrotic sections were immunostained for the neutrophil marker Ly6G, MPO, cleaved caspase-3 and proliferation marker Ki-67 (40×); (**E**) Tumor infiltrating neutrophils were elevated in MPO^–/–^ mice compared to WT and KYC treated mice.

At the same time, separate groups of wild type mice were treated with or without KYC (sub-cu, daily, 3.0 mg/kg) for the duration of the experiment. The size of the tumor graft was evaluated with Vernier calipers and volume calculated accordingly. MPO^–/–^ mice had significantly reduced tumor volume (*p* < 0.05) than mice in the control group, while those treated with KYC were indistinguishable from the control group (Figure 4A). Tumors were excised and weighed on day 17, and the tumor weight for MPO^–/–^ mice was 3 times less than control mice (*p* < 0.01) (Figure 4B). However, tumors from mice treated with KYC at the dose given were indistinguishable in size from the control group (Figure 4B). H&E staining indicated increased areas of necrosis in tumors engrafted in MPO^–/–^ mice (Figure 4C) in comparison to wild type mice or those treated with KYC. These necrotic areas also stained strongly for Ly6G, MPO and cleaved caspase-3. A higher magnification analysis of non-necrotic tissue sections indicated increased number of tumor infiltrating neutrophils (Ly6G^+^), with no obvious changes in tumor cell proliferation (Ki-67^+^) (Figure 4D,E).

### 2.5. Discussion

We have previously observed that BHT administration induced neutrophil infiltration in the airways of mice, and that neutrophils were required for BHT-induced promotion of lung carcinogenesis [19]. Lung neutrophil infiltration is consistent with observations using other mouse lung tumor promoters including lipopolysaccharide (LPS) and non-typeable *Haemophilus influenza* lysate (NTHil), which are often used to model COPD [53,54,55]. We have now established that BHT induces an increase in extracellular MPO in the BALF of mice and inhibition of MPO activity reduces inflammatory promotion of lung carcinogenesis. This is consistent with recent data where MPO protein levels were significantly increased in the BALF of patients with COPD and lung cancer [50]. 

Our study is the first to report that inhibition of MPO activity with a non-toxic biologic tripeptide, inhibited tumor burden. KYC is novel and a recently described MPO activity inhibitor [51,52]. The tyrosine is a natural substrate of MPO and serves as a scavenger for activated MPO preventing its ability to convert H_2_O_2_ to HOCl. The adjacent cysteine scavenges these tyrosyl radicals, reacts with glutathione *in vivo* and regenerates KYC. Studies have shown that KYC: (a) inhibits MPO-mediated oxidant production *in vitro* and *in vivo*, (b) is non-toxic in mice, and (c) daily administration to mice increases vascular function [51,52]. 

KYC reduced MCA-initiated BHT-promoted lung tumor multiplicity by 50% in BALB mice, without affecting tumor diameter. Interestingly, KYC at a 10-fold higher dose (3 mg/kg/d) had no effect on the growth of LLC tumor cells engrafted on the flank of B6 mice. In contrast, mice genetically deficient in MPO had significantly smaller tumors, as measured by weight and volume. This may be explained by either: (*i*) limited bioavailability of KYC in the LLC tumor graft resulting in limited MPO activity inhibition, as compared to the complete ablation of MPO activity in the MPO^–/–^ mice; or (*ii*) that MPO activity only plays a role in the early phases of cancer cell growth such as initiation/promotion, which are not modeled in the LLC tumor cell graft system. This might suggest that MPO protein (independent of activity) could be sufficient for subsequent tumor growth and survival. While others have observed reduced asbestos-induced lung epithelial cell proliferation in MPO-deficient mice [56], the engrafted tumors do not appear to proliferate less in the knockout. We however did observe significant necrosis in tumors in knockout mice, and this was co-incident with strong staining for neutrophils, MPO and the apoptosis marker, cleaved caspase-3. This is particularly noteworthy as a previous study in ovarian cancer cells lines demonstrated that tumor cell generated MPO, in conjunction with iNOS, induced S-nitrosylation of caspase-3, thus protecting cells from apoptosis [42]. NO^+^ generated by MPO can nitrosylate and inactivate caspase-3, via nitrosylation of thiol groups (S-nitrosylation), ultimately protecting transformed cells against apoptosis. Although LLC cells do not express MPO, this mechanism of MPO-mediated inhibition of caspase-3 activity could plausibly operate because MPO can enter lung epithelial cells [47]. In non-necrotic regions of the tumor, proliferation (Ki-67 staining) was not affected. Immunohistochemical staining in these regions also showed that MPO knockout mice exhibited increased neutrophil numbers in the tumor compared to WT mice. This may suggest that under normal conditions, MPO activity suppresses neutrophil tumor infiltration, or that a non-enzymatic function of MPO is required for tumor growth post-initiation. Previous studies have indicated that enzymatically inactive MPO can play a critical role in modulating immune responses including release of pro-inflammatory cytokines, aiding in leukocyte extravasation and enhancing neutrophil survival [57,58,59,60]. Thus, activity-independent functions of MPO post-initiation may play a pivotal role in shaping the immune response to alter tumor growth. 

MPO may also play a larger role in processes occurring immediately after tumor initiation by carcinogen. Direct activation of polycyclic aromatic hydrocarbons into DNA damaging metabolites by MPO has been documented [61]. Although we did not measure the effects of BHT and KYC treatment on MCA-DNA adduct formation, this could be a focus of future investigation. A previous study demonstrated that after six weeks of BHT administration, the composition of lung immune cell infiltrates is changed to high lymphocyte and macrophage populations, but few neutrophils [15,62]. Therefore, it is conceivable that the pro-tumor effects of neutrophil MPO may occur in the window immediately post tumor initiation. 

Various models of inflammation-promoted tumorigenesis have demonstrated that neutrophils play a key aspect in the progression of cancer. A recent study in a oncogenic K-Ras murine model of NTHil-promoted lung cancer demonstrated that either depleting neutrophils or blocking neutrophil migration via the KC/Cxcl1 receptor, Cxcr2, reduced tumor burden [63]. Our study builds upon these observations and demonstrates that MPO is an important mediator of inflammatory-promoted tumorigenesis in the lung. Recent studies have also established that another primary granule enzyme, neutrophil elastase (NE), is critical for lung cancer cell growth [63,64]. Neutrophils secrete low levels of NE that enter lung epithelial cells and affect proliferative and survival signaling pathways. Interestingly, MPO can also enter lung epithelial cells [47,65]. The influence of MPO on the pro-tumor functions of NE, or the synergism between the two enzymes has not been investigated.

KYC may prove to be a promising preventive agent in individuals who suffer from chronic inflammation linked to high risk of lung cancer. Our data strongly supports the pursuit of MPO inhibitors as potential chemopreventive agents, and evaluation in other lung cancer prevention models should be considered. In a guinea pig model of smoking induced COPD, administration of a small molecule 2-thioxanthine MPO inhibitor prevented development and progression of emphysema and small airway remodeling [66]. 

While MPO is involved in innate defense against pathogens, administration to humans in a preventive atmosphere is unlikely to be immunosuppressive. An estimated 0.1% of the population has some degree of MPO deficiency, is asymptomatic and exhibits no susceptibility to severe or persistent infection [67,68]. This suggests that neutrophils have multiple redundant defense mechanisms and MPO inhibitors would not inevitably compromise immunity. Other MPO inhibitors, such as azides, hydrazides, and hydroxamic acids covalently bind the MPO heme site and act as suicide substrates for the enzyme. These inhibitors are not as beneficial as KYC, because they have off-target effects and are toxic, thus reducing their probability of being used in a chemopreventive setting [39]. KYC is a promising preventive agent with a unique mechanism of action and relatively favorable toxicity profile [51,52].

## 3. Experimental

### 3.1. BHT Treatment and BALF Collection

Inbred female BALB/cByJ (BALB) mice were acquired from the Jackson Laboratory (Bar Harbor, ME, USA). For lung inflammation assays, mice (at 7 weeks of age) were injected i.p. with 300 mg/kg body weight of BHT dissolved in 0.2 mL Mazola corn oil. Corn oil alone was used as a vehicle control. Three days after the final injection, bronchoalveolar lavage fluid (BALF) was collected as previously described [19]. Cells were collected by centrifugation (10 min @ 200g) and supernatant was used for determination of myeloperoxidase protein and activity. KYC, *N*-acetyl lysyltyrosylcysteine amide, was synthesized by Biomatik (Cambridge, ON, Canada) using solid phase technique with Fmoc [*N*-(9-fluorenyl) methoxycarbonyl] chemistry and purified with HPLC to 98% purity. All animal procedures were approved by The Medical College of Wisconsin Animal Care and Use Committee.

### 3.2. Flow Cytometry Analysis

Cells isolated from BAL were prepared for flow cytometry as previously described [19,69]. All antibodies (Gr-1 (RB6-8C5), Ly6G (1A8) and isotype control antibodies) were acquired from BD Pharmingen (San Diego, CA, USA) or eBioscience (San Diego, CA, USA) conjugated to either fluorescein isothiocyanate (FITC) or allophycocyanin (APC). Flow cytometry was performed using a BD LSRII flow cytometer at the Flow Cytometry Core at the Blood Research Institute (Milwaukee, WI, USA).

### 3.3. CXCL1/KC ELISA

CXCL1/KC levels in the BALF were determined using a mouse CXCL1/KC sandwich ELISA from R&D Systems (Minneapolis, MN, USA). ELISA was performed per manufacturer’s directions. Briefly, 50 µL of samples or standard amounts of recombinant KC were added to each well and incubated at room temperature for two hours. After five washes, 100 µL of a polyclonal KC antibody conjugated to horshradish peroxidase was added to each well and incubated for two hours at room temperature. After another five washes, a 100 µL of the substrate solution, hydrogen peroxide and tetramethylbenzidine, was added and incubated in the dark for 30 min. A 100 µL stop solution, diluted hydrochloric acid, was added and read at 450 nm with a correction wavelength at 540 nm on the Tecan Infinate M200 Pro plate reader.

### 3.4. MPO ELISA and Activity Analysis

MPO protein levels in the cell-free fraction of the BALF were determined using a mouse MPO sandwich ELISA by Hycult Biotech (Uden, The Netherlands). ELISA was performed per the manufacturer’s directions. Activity of MPO in BALF was determined by the reduction of *o*-dianisidine. Assay buffer consisted of 25 mM phosphate buffer pH 6.5, 0.5 mM *o-*dianisidine dihydrochloride (Sigma Aldrich, St. Louis, MO, USA) and 100 µM H_2_O_2_ (Sigma Aldrich). 20 µL of BALF sample was mixed with 200 µL assay buffer and read at 460 nm on a Tecan Infinite M200 Pro plate reader (Tecan Systems, San Jose, CA, USA) for ten minutes as previously described [70,71]. 

### 3.5. Two Step MCA/BHT Carcinogenesis Assays

A single i.p. injection of MCA (25 mg/kg body weight in corn oil) was followed by 6 weekly i.p. injections of BHT (1st injection: 150 mg/kg, 2nd–6th injections: 200 mg/kg). KYC (0.3 mg/kg) peptide dissolved in PBS, was given sub.cu daily beginning 4 days post MCA, and ending 1 week after the final BHT injection. At endpoint (20 weeks after MCA injection), mice were anesthetized and euthanized. The thoracic cavity was opened, trachea cannulated, and lungs inflation fixed and excised as previously described [19]. Lobes were separated and surface lung tumor number/multiplicity, diameter (min. 0.25 mm measured by vernier calipers) were blindly assessed under a microscope. 

### 3.6. Lewis Lung Carcinoma Cell Heterotopic Syngeneic Graft Model

1 × 10^6^ Lewis Lung Carcinoma (LLC) cells in 0.2 mL of media (DMEM +10% FBS) were injected into the right flank of female (7 weeks, n = 6 per group) C57BL/6J and C57BL/6J-MPO^–/–^ deficient mice (Jackson Labs #4265). WT mice were treated daily with 3 mg/kg KYC (subcutaneous) or with PBS controls beginning on day 1 until endpoint. Tumor volume (V = (length × width^2^)/2) was monitored via caliper measurements over 17 days and on the final day excised tumors were weighed.

### 3.7. Immunohistochemical Staining

Tissues were fixed for 24 h in zinc formalin then placed in 70% ethanol. Tissue samples were processed in paraffin by the Children’s Research Institute Histology Core. Antigen retrieval was done in citrate buffer at pH 6.0 for 20 min. Sections were stained using a DAKO Autostainer Plus (DAKO North America, Carpinteria, CA, USA) at dilutions of 1:200 for anti-Ly6G (NIMP-R14, Santa Cruz Biotechnology, Dallas, TX, USA), 1:150 for anti-MPO (ab9535, Abcam, Cambridge, MA, USA), 1:100 for cleaved caspase-3 (CP229B, Biocare Medical, Concord, CA, USA), 1:200 for Ki-67 (D3B5, Cell Signaling Technology, Danvers, MA, USA) and for H&E. Slides were imaged on a Nikon Eclipse 50*i* microscope (Nikon Instruments, Melville, NY, USA) and analyzed using NIS Elements BR software (Nikon). Quantification was done by selecting cells that stained positive for the antibody of interest, selecting a threshold, which was then applied to all images in that set. The percent area was calculated by determining the area that stained positive for the antigen and dividing it by the total area of the image. 

### 3.8. Western Blot Analysis

Tumors were excised and frozen at –80 °C until needed. Tumor tissue was mechanically homogenized in RIPA lysis buffer consisting 1 mM 2-mercaptoethanol, phosphatase inhibitor cocktails II and III (Sigma Aldrich), and Sigmafast protease inhibitors (Sigma Aldrich). Samples were centrifuged at high speed and the supernatant protein concentration was determined using Peirce BSA Protein Assay Kit (Thermo Scientific, Rockford, IL, USA). 10 µg of protein was loaded into a 4%–15% mini-Protean TGX polyacrylamide gel (Bio-Rad, Hercules, CA, USA) and electrophoresed at 180 volts. Protein was then transferred to PVDF at 100 volts for an hour and then blocked for 30 min in 1% milk in TBST buffer. Primary antibody (1:3000 dilution for cleaved caspase-3 (Cell Signaling, 5A1E) and 1:15,000 for actin (A2066, Sigma Aldrich) was incubated with the membrane overnight. The next day, secondary HRP-conjugated antibody was added for 1 hour, and blots were developed using SuperSignal West Femto Maximum Sensitivity Substrate (Thermo Scientific, Rockford, IL, USA) and exposed to film.

### 3.9. Statistical Analysis

All analysis of significance (*p* < 0.05) was performed by two-tailed Student’s *t*-Test. Wilcoxon test was performed for MCA/BHT/KYC tumor study. 

## 4. Conclusions

In this study, we demonstrated that inhibition of MPO activity by a novel tripeptide inhibitor KYC in the MCA-initiated BHT-promoted model of lung carcinogenesis, reduced tumor burden by 50%. Interestingly, treatment with KYC in an LLC tumor graft model exhibited no effect on tumor size, yet tumor size was significantly reduced in MPO^–/–^ mice. We postulate that MPO activity is required during the early phases of tumor initiation and promotion, while non-enzymatic functions of MPO play a role during later phases of tumor progression through protection of cancer cells against caspase-3 mediated cell death. Patients at increased risk of lung cancer, such as those with COPD, may benefit from prophylactic treatment with non-toxic MPO inhibitors to reduce chances of developing lung cancer. 
